# Experience of observation skill workshop intervention for ophthalmologists in fellowship training

**DOI:** 10.12688/f1000research.148008.2

**Published:** 2024-10-07

**Authors:** Snigdha Snigdha, Avinash Pathengay, Anamika Patel, Shefali Pandey, Shobha Mocherla, Ruby Kala Prakasam

**Affiliations:** 1 Academy for Eye Care Education, L V Prasad Eye Institute, Hyderabad, Telangana, 500034, India

**Keywords:** Observation skill, Communication skill, Ophthalmology education, Workshop intervention, Self-rating questionnaire

## Abstract

**Background:**

To gauge the impact of an interventional workshop conducted to measure the observation skills of 34 postgraduates during induction into an ophthalmology fellowship.

**Methods:**

A seven-hour workshop was conducted with the ophthalmology trainees. Trainees from the 2022 batch of ophthalmology fellowships (21 females and 13 males) were included in the study. The pre-workshop assessment comprised two non-clinical images to spot the difference and five clinical images from various subspecialties of ophthalmology. This was followed by workshop and Post workshop assessment. The pre- and post-observation grades of participants were then compared by masked ophthalmologists. The Wilcoxon signed-rank test was used to compare scores at two time points, with a p-value < 0.05 for statistical significance.

**Results:**

Statistical analysis revealed that the observation skill score was significantly higher after the workshop intervention (M _d_ = 4.00, n = 34) compared to the pre-workshop score (M _d_ = 1.85, n = 34), p-value = 0.000.

**Conclusions:**

Workshops on specific/selected foundational skills, such as observation skills and communication skills, must be integrated into the curricula of basic medical degree and specialty medicine to equip medical professionals with attentive observation and deep learning.

## Practice points

• Observation is a core skill in the repertoire of foundational skills that enhance medical professionalism, making clinicians good collaborators and communicators with peers and patients.
• Structured training in observational skills could help to improve clinical observation and related clinical diagnoses, sensitizing clinicians to the functional vulnerability of patients, and hastening collaborative decision making, treatment plans, and patient outcomes.

## Introduction

Observation refers to gathering sensorial information, and learning refers to making sense of these observations based on previous knowledge, discerning (the commonalities and differences), deducing (combing and appropriately reacting to the input received), and differentiating (the rope from a snake). Such observational learning helps form perceptions and improve functioning. ‘Observation skill’ is a quintessential foundational ‘skill,’ requiring its cultivation and refinement from the moment one enters the medical profession. In medicine, observational skills are fundamental to developing competencies, such as clinical reasoning and interpretation. As observation is a core art skill, exposure to the art in medical schools demonstrably paves the way for the development of enhanced visual perception, observation skills, and empathy. ^1^ Being introduced to spirituality in medical education leads to students’ intrapersonal development, health, and genial responsiveness. ^2^ The depth of perception quickly rises above mere superfluous assessment to a oneness with the observed that takes the observer’s breath away. Training in observational skills is required to develop ability, depth, and consistency. Studies have shown that crucial details can be missed even when observing closely, prompting the need for a flexible and adaptable approach to training oneself and others in observation skills. ^3^

Throughout history, honing one’s observational skills has been emphasized in the medical sciences, recognizing its fundamental role in clinical medicine and applicable across all specialties. Regrettably, observation is frequently assumed and, despite its significance, marginalized in today’s world. ^4^ Being linked to critical thinking, the capacity to observe is venerated to provide a spot-on solution to a problem. We know that observation effectively, efficiently, and responsibly resolves an issue that begins by identifying the problem early. Observation skill helps us to develop a keen eye for detail, familiarity with the nomenclature, procedure and technique, going-by-the-book recognition and cross verification, with curious intention and implementation ‘using peripheral vision and subsidiary awareness’. ^5^ Taking a leaf from some medical schools that have successfully integrated art into their teaching approach for skill development in observation, our team of educators designed workshops to train ophthalmology trainees at the fellowship level in much-needed observation skills. We share our experiences with the outcomes of an observation skill enhancement workshop.

## Methods

### Observation skill workshop: The genesis

The Education Academy recognized some essential foundation skills for good clinical practice, with observation skill as one of these. The seven-hour physical workshop was conducted on the first of July 2022 for post-graduate fellows at the institute. Learnings acquired from virtual workshops held one year earlier helped us improve the structure and quality of the present physical workshop. The data captured through this workshop were reviewed retrospectively. This study complied with the tenets of the Declaration of Helsinki. The Study involved study participants’ perceptions, and study approval was obtained from the Institutional Review Board (IRB) of the Institute Ethics Committee (IEC) Hyderabad Eye Research Foundation. Ethics Reference Number: LEC-BHR -01-23-151 was approved on 22 ^nd^ March 2023.

The details are given below.

Post-graduate fellows (Batch 2022, N=34) were invited via e-mail to participate in the foundation skill workshops. The e-mail communication included clear guidelines for the fellows to bring their own laptop/tablet device to fill the pre- and post-workshop assessment. The actual workshop started with an introductory speech by the facilitator/moderator explaining the structure and content of the workshop and the program schedule. The introduction included information regarding the research component of the workshop and invited participants to participate in the workshop and the pre-post assessment.

The pre-workshop assessment comprised two non-clinical images to spot the difference and five clinical images from various subspecialties of ophthalmology, such as medical retina, uveitis, cornea, oculoplasty, and strabismus. The pre-workshop assessment of trainees was conducted using Quizizz, an online gamified student engagement platform. Each image was projected for five minutes, and participants’ responses were obtained. The total duration was 35 minutes, and the responses were stored in the Quizizz application and later retrieved. Clinical images were graded by two expert ophthalmologists who were blinded to the intended use.

After the pre-workshop assessment, the facilitator/moderator made a presentation on observation as a preeminent foundational skill, dwelling on its importance in daily practice in the clinical setting and in academic research. Such interventions have been made in the past in ophthalmology and various fields of medicine. The entire workshop was interactive, piquing audience interest by showcasing images and then inviting all trainees to comment on and add points to the description of the image if they considered that information was inaccurate or insufficient. After attending the workshop, participants were asked to form four groups and take a tour of the campus where various photographs and paintings were displayed that riveted their attention. The trainees were encouraged to describe their observation points by looking at random sketches/paintings, or photographs. The facilitator/moderator further guided the groups while making collective observations. In the last segment of this workshop, the participants were assessed on the Quizizz platform for their observation skills, using a new set of two non-clinical images and five clinical images.

At the end of the workshop, the participants’ reflections were collected after committing to confidentiality and anonymity. The pre- and post-observation grades of participants were then compared.

### Statistical analysis

Analyses were performed using SPSS Version 21.0 (IBM SPSS Statistics Inc., Armonk, NY, USA) software. ^6^ Normality was checked using the Shapiro–Wilk test. Continuous data are presented as median or interquartile range. Categorical data are presented as proportions. Comparative analyses were performed using the Wilcoxon signed-rank test, and a probability value < 0.05 was considered statistically significant.

## Results

**Figure 1.**
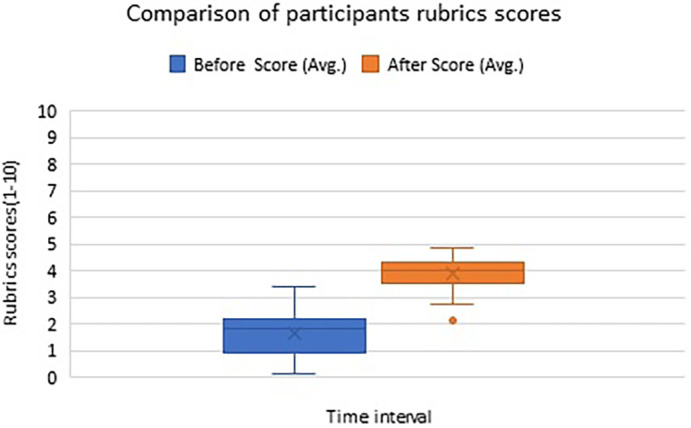
Box-Whisker Plot illustrating participants’ average score about attribute ‘observation’ skill acquisition on a scale of 1-10 at two timepoints – before and after the workshop.

Analyses of the average rubric scores of 34 first-year ophthalmology fellows {21 (62%) females and 13 (38%) males} training at two different time points are illustrated in the box-whisker plot in Figure 1. ^15^ The mean age of the participants was 31.2 ± 3.7 (Mean, SD) years.

The Wilcoxon signed-rank test revealed that the score for observation skill was significantly higher after the workshop intervention (M _d_ = 4.00, n = 34) compared to the pre-workshop score (M _d_ = 1.85, n = 34), p-value = 0.000. Box-Whisker Plot illustrating participants’ average scores about attribute ‘observation’ skill acquisition on a scale of 1-10 at two timepoints – before and after the workshop shows improvement in observation skills scores after attending the workshop ( Figure 1).

Overall, our study indicates that our observation skill workshop positively affected ophthalmology trainees.

## Discussion

Observation is the predominant way of learning for a significant proportion of undergraduate and post-graduate training in medical education. However, it is difficult to ascertain a template for observation skill training that can be applied in all learning situations. While it is seen that the exposure to observation skill training can be largely standardized, the outcomes vary. This intractability may be because much of what is learned is as imaginary mental maps, building on the foundations created by our past exposure, learning, and recall, all of which vary among individuals.

Constructivism, a key principle of learning in which both trainees and educators create new knowledge by building on their existing information, has a significant impact on clinical practice. This new knowledge, born out of teacher-trainee synergy and team spirit, empowers medical educators and their trainees to uncover the unknown. ^7^

Training courses have been developed in the past to transfer much-needed observation skills to medical students. Unfortunately, lacunae are evident in the transfer of observation skills. There are several reasons why observation skills are not formally taught in medical schools. One reason is that the capacity to observe is often seen as a ‘soft skill,’ which is not as important as technical skills. Another reason is the commonly held belief that observation skills are directly associated with individual cognition and response and cannot be systematized or formulated. Another reason is that, due to the complexity of observation and its elusiveness, the assessment of observation skill cannot be entirely standardized.

In 2020, we began to actively teach observation skills to our trainees. The COVID19 lockdowns gave us an opportunity to explore deep learning and look at our current challenges, and that is when we planned to focus on teaching comprehensive clinical observation skills, integrating it into the formal course curriculum by conducting workshops during the ophthalmology fellowship induction program. This was further propagated in various learning forums when learners were encouraged to collaborate to make valid observations in greater quantity and interpret their observations in chronological order. Providing trainees with opportunities to practice their observation skills can be a good start to help them enhance their own understanding of various aspects of observation and practice. Feedback from peers and educators can help trainees significantly improve their observation skills within a set time frame.

### Interplay of Art and Science

Integrating art and medicine, as demonstrated in the collaboration between a medical school and an art museum, promises to enhance the observation, description, and interpretation skills of medical students. ^8^ Ongoing unlearning and relearning leads to clarity in our perceptions, conquering setbacks, leading to newer ways of doing something, and eventually, distinctive new knowledge. Authors adhering to traditional scholasticism have endeavored to preserve this art, challenging its decline by reinvigorating observation skills through unique methods, much like an art form. ^9^

Research highlights that clinical methods excel in pattern recognition, whereas art-based approaches uniquely foster emotional recognition, empathy, narrative understanding, and a multifaceted perspective, which extends to healthcare. ^10^ Concepts like the spider web narrative and the Golden Minute underscore the importance of trainees honing their observation skills and attending to subtle patient details. ^11^ Moreover, integrating visual art training into medical education, primarily focusing on preclinical students, addresses various skills, such as observation, empathy, and teamwork, using established methods, such as visual thinking strategies or artistic thinking. ^12^ Further, a study in ophthalmology demonstrates that formal art observation training significantly improves observation skills in medical students, with potential applications in specialized medical fields. ^13^

### Conclusion – Wanted: A purposeful leap of faith

Just as an owl can easily see what is at the back of its head by flexing its neck three-quarters on both sides, the observant nature of a Master of Cognition (known in India as an Ashtavadhani) permits a speedy and appropriate response to difficult questions from multiple directions with great nonchalance. However, how can one observe which is invisible, inaudible, imperceptible, unknowable, or beyond the horizon, reach, or ken? Historically, humankind has willed the individual and collective consciousness to access power beyond what is known (hailing discovery, invention, innovation, wait and watch, intuition, extra-sensory perception, serendipity, and so on). Again, the purposeful leap of faith that helped outer space practitioners of our cosmos land a rover nearest to the unknown south side of the moon may well be the prescription for us to bring into the known great and small mysteries closer home waiting to be noticed, studied, and unraveled by the observant eye.

Observation skills are important for medical professionals to initiate self-efficacy in the care setting, benefiting their patients and themselves in achieving self-dependence and self-care. At the meso level, these help to resolve health issues for the community using multimodal preventive and promotive care. At the macro level, clinicians provide curative, rehabilitative, or palliative care, collated and systematized based on long, hard, sensitive, and personal observation. At the micro level, the good doctor’s sensitivity in the doctor-patient interaction helps them to be aware of their own biases which prevent the doctor from providing care that would make the patient self-dependent. ^14^

Being observant is essential for success in many areas of life including education, work, and relationships. The importance of observation skills is speedily sizing up, facilitating process improvement, identifying a concern early (before it becomes a problem), and resolving knotty problems. At the tertiary level, a guided approach to enhance one’s observation skill and arrive at a logical interpretation can help doctors make more accurate clinical diagnoses. However, as clinical knowledge cannot in isolation serve patients, medical professionals must also readily impart new learning about the theory and practice of observation skills, actively letting go of inherent biases. By sharing their clinical prowess with their peers, superiors, and trainees in other specialties of medicine, they can become better collaborators and enhance their professionalism. Similarly, through observant empathy of functional vulnerability, such as vision loss in patients (their significant others), medical professionals can practice providing better care to the human being in every patient, collaborating without exception.

## Ethics and consent

Study approval was obtained from the Institutional Review Board (IRB) of the Institute Ethics Committee (IEC) Hyderabad Eye Research Foundation. Ethics Reference Number: LEC-BHR -01-23-151 was approved on 22 ^nd^ March 2023. The seven-hour physical workshop was conducted on the 1st of July 2022 for post-graduate fellows at the institute. Ethical approval was taken at a later date as we were unaware that we would publish the results from the workshop. The success of the workshop encouraged us to evaluate the retrospective data collected from the workshop and write a manuscript, therefore we needed to seek ethical approval to move forward and with formal manuscript writing. This study complied with the tenets of the Declaration of Helsinki.

Informed consent was taken verbally in the beginning of the workshop. Taking verbal consent was approved by the ethics committee. The workshop instructor then explained the entire workshop in a stepwise manner. On the research aspect, the instructor circulated an online questionnaire link for data capture with the following remarks as a part of the link “You are invited to participate in a research study on “observation skills”. The purpose of the study is to capture the perceptions of ophthalmology fellows on the importance of “observation skills” for clinical management. Please take a few moments and complete this survey by clicking on Next page. By completing this survey, you are consenting to participate in this study.”

## Data Availability

Figshare: Observational Skills data. https://doi.org/10.6084/m9.figshare.25138505.v1. ^15^ Data are available under the terms of the Creative Commons Zero “No rights reserved” data waiver (CC0 1.0 Public domain dedication). Figshare: OSW images reference document.pdf, https://doi.org/10.6084/m9.figshare.26861617.v1. ^16^ Figshare: OSW-QD additional data.xlsx https://doi.org/10.6084/m9.figshare.26869246.v1. ^17^ Data are available under the terms of the Creative Commons Attribution 4.0 International license (CC BY 4.0).
