# Supplementary material for: Experience of observation skill workshop intervention for ophthalmologists in fellowship training
Source: F1000Res. 2024 Oct 7;13:524. Originally published 2024 May 22. [Version 2] doi: 10.12688/f1000research.148008.2 (PMC11484534; doi:10.12688/f1000research.148008.2)
Supplement: Peer review history

# Reviewer report: Reviewer response for version 2

**DOI:** 10.5256/f1000research.171975.r329566

**Published:** 2024-10-16

**Authors:** Purvi Bhagat

**Affiliations:** 1 B.J. Medical College and Civil Hospital, Ahmedabad, Gujarat, India

The responses provided by the author are satisfactory. No more comments.

Is the work clearly and accurately presented and does it cite the current literature?

Yes

If applicable, is the statistical analysis and its interpretation appropriate?

Yes

Are all the source data underlying the results available to ensure full reproducibility?

Partly

Is the study design appropriate and is the work technically sound?

Yes

Are the conclusions drawn adequately supported by the results?

Yes

Are sufficient details of methods and analysis provided to allow replication by others?

Partly

Reviewer Expertise:

Ophthalmology, Glaucoma, Medical Education

I confirm that I have read this submission and believe that I have an appropriate level of expertise to confirm that it is of an acceptable scientific standard.

# Reviewer report: Reviewer response for version 1

**DOI:** 10.5256/f1000research.162270.r303770

**Published:** 2024-08-08

**Authors:** Helena Prior Filipe

**Affiliations:** 1 West Lisbon Hospitals Center, Lisbon, Portugal

Please accept my appreciation for the opportunity to review the manuscript that discusses the impact of an interactive educational program aimed at enhancing observational skills at the fellowship level in ophthalmology.

 The abstract provides a clear summary of the manuscript. The use of the term "foundational skills" instead of the more common "soft skills," deserves emphasis as it acknowledges the significance of skills beyond medical expertise in defining the identity of a healthcare professional.

 The keyword selection is strategic, and the practice points effectively convey the main research messages.

 The manuscript is well-structured and consistently presents information. The research methods and results are appropriately described and supported by statistical analysis, and the discussion is comprehensive in its reflection on the impact of improved observation skills on communication, collaboration, and professionalism.

 The study is based on an online closed-answer survey. In future work, the authors might consider incorporating semi-structured interviews to gather more comprehensive data using a mixed methods approach. Additionally, further evaluation of the intervention's impact would be valuable, considering the organization and its potential effects on faculty, retention fellows preference, the hidden curriculum, organizational culture, and 360º feedback.

 The authors might consider detailed information about the educational delivery methods utilized in the workshop, including the implementation of educational science strategies, eg. visual thinking strategies  [ ref 1 ] and specific methods of social and active learning. The manuscript raises important questions about the changing landscape of medical practice and the evolving role of foundational skills.

 The authors could potentially explore cultural competence in teaching and practicing ophthalmology, particularly in the context of Western and Eastern cultural differences [ ref 2 ]. Moreover, they may continue investigating how foundational skills can be effectively learned, practiced, and assessed to impact wellbeing and patient care positively.

 In conclusion, kudos to the authors for their research in this vital area to deliver high-quality eye care, focusing on a visually engaging specialty such as ophthalmology.

Is the work clearly and accurately presented and does it cite the current literature?

Yes

If applicable, is the statistical analysis and its interpretation appropriate?

Yes

Are all the source data underlying the results available to ensure full reproducibility?

Yes

Is the study design appropriate and is the work technically sound?

Yes

Are the conclusions drawn adequately supported by the results?

Yes

Are sufficient details of methods and analysis provided to allow replication by others?

Partly

Reviewer Expertise:

Ophthalmology, healyth professions education

I confirm that I have read this submission and believe that I have an appropriate level of expertise to confirm that it is of an acceptable scientific standard.

## References

[1] Reference source

[2] : Connecting West and East. *International Journal of Molecular Sciences* .2019;20(9) : 10.3390/ijms20092333 10.3390/ijms20092333 PMC654057931083489

# Reviewer report: Reviewer response for version 1

**DOI:** 10.5256/f1000research.162270.r303776

**Published:** 2024-08-08

**Authors:** Purvi Bhagat

**Affiliations:** 1 B.J. Medical College and Civil Hospital, Ahmedabad, Gujarat, India

The article highlights the importance of including subtle skills like observation, communication and critical thinking in the clinical training of postgraduate students. The article is written well but certain important aspects of the methodology need mention and elaboration.

 1. Validation of the pre and post assessment including the images needs to be mentioned. Also, who were the validators and what was the type of validation done - face, content etc.?

 2. The images form the core of the study. These need to be shared.

 3. The effect of a one day 7-hour workshop may not be transferable to similar observation skills in clinical / patient care. An added assessment through OSCE or Mini CEX would have been more relevant.

 4.The qualitative analysis of the reflections is not mentioned. Needs to be added.

 5.The study addresses only the first level of the Kirkpatrick. Utility is when the higher levels are achieved.

Is the work clearly and accurately presented and does it cite the current literature?

Yes

If applicable, is the statistical analysis and its interpretation appropriate?

Yes

Are all the source data underlying the results available to ensure full reproducibility?

Partly

Is the study design appropriate and is the work technically sound?

Yes

Are the conclusions drawn adequately supported by the results?

Yes

Are sufficient details of methods and analysis provided to allow replication by others?

Partly

Reviewer Expertise:

Ophthalmology, Glaucoma, Medical Education

I confirm that I have read this submission and believe that I have an appropriate level of expertise to confirm that it is of an acceptable scientific standard, however I have significant reservations, as outlined above.

# Response

**Published:** 2024-08-29

**Authors:** Snigdha Snigdha

**Affiliations:** Academy for Eye care Education, L V Prasad Eye Institute, Hyderabad, Telangana, India

Dear Dr Bhagat

 Thank you for spending your precious time and effort it reviewing our manuscript. We have tried our best to answer/make amends as suggested.

**1. Validation of the pre and post assessment including the images needs to be mentioned. Also, who were the validators and what was the type of validation done - face, content etc.?** Thank you for the question. A panel of five ophthalmologists, each specializing in a distinct subspecialty, were convened to assess images. These experts conducted a comprehensive analysis of each image, identifying key observation points that were used to develop a scoring rubric. The same was then applied to both pre- and post-assessment data, enabling a direct comparison of participant performance. This validation approach ensured that the assessment instruments were aligned with current ophthalmic standards and provided a robust foundation for evaluating the effectiveness of the workshop. We have added the images as an addition supplement to this paper.

**2. The images form the core of the study. These need to be shared.**

 We have added this data: https://doi.org/10.6084/m9.figshare.26861617.v1

**3. The effect of a one day 7-hour workshop may not be transferable to similar observation skills in clinical / patient care. An added assessment through OSCE or Mini CEX would have been more relevant.**

  We accept you suggestion. We will certainly add this additional assessment in our future workshops and studies.

**4. The qualitative analysis of the reflections is not mentioned. Needs to be added.**

  We have added this data: https://doi.org/10.6084/m9.figshare.26869246.v1

**5. The study addresses only the first level of the Kirkpatrick. Utility is when the higher levels are achieved.**

  Thank you for the valuable feedback. Your observation regarding the study's focus on the first level of the Kirkpatrick model is well-noted. We acknowledge the importance of achieving higher-level outcomes, such as utility, in our educational endeavors. It is very crucial for us to implement a certain pedagogy which further reinforces the learnings from this workshop which is provided during our immersive induction program. We have integrated this exercise of manifesting observations and correlating them in clinical scenarios as a regular lesson plan in most of our academic sessions.
